# Horizontal Gene Transfer and Redundancy of Tryptophan Biosynthetic Enzymes in Dinotoms

**DOI:** 10.1093/gbe/evu014

**Published:** 2014-01-21

**Authors:** Behzad Imanian, Patrick J. Keeling

**Affiliations:** Department of Botany, Canadian Institute for Advanced Research, University of British Columbia, Vancouver, British Columbia, Canada

**Keywords:** tryptophan biosynthesis, dinotoms, tertiary endosymbiosis, biochemical redundancy, dinoflagellates, diatoms

## Abstract

A tertiary endosymbiosis between a dinoflagellate host and diatom endosymbiont gave rise to “dinotoms,” cells with a unique nuclear and mitochondrial redundancy derived from two evolutionarily distinct eukaryotic lineages. To examine how this unique redundancy might have affected the evolution of metabolic systems, we investigated the transcription of genes involved in biosynthesis of the amino acid tryptophan in three species, *Durinskia baltica*, *Kryptoperidinium foliaceum*, and *Glenodinium foliaceum*. From transcriptome sequence data, we recovered two distinct sets of protein-coding transcripts covering the entire tryptophan biosynthetic pathway. Phylogenetic analyses suggest a diatom origin for one set of the proteins, which we infer to be expressed in the endosymbiont, and that the other arose from multiple horizontal gene transfer events to the dinoflagellate ancestor of the host lineage. This is the first indication that these cells retain redundant sets of transcripts and likely metabolic pathways for the biosynthesis of small molecules and extend their redundancy to their two distinct nuclear genomes.

## Introduction

The primary endosymbiosis with a cyanobacterium that gave rise to the plastids found in glaucophytes, red algae, green algae, and plants was the prelude for the subsequent rounds of endosymbioses. Many eukaryotes independently acquired their plastids through secondary endosymbioses with either a green or red alga (Archibald and Keeling 2002; Palmer 2003; Keeling 2010, 2013). In a third round of endosymbiosis, new dinoflagellate hosts took up certain algae with secondary plastids, which later reduced to different degrees. In Kareniaceans and perhaps Dinophysis, for example, their respective haptophyte and cryptophyte endosymbionts were reduced to just the plastid (Patron et al. 2006; Garcia-Cuetos et al. 2010).

This genetic and morphological reduction of the endosymbiont was accompanied by large-scale gene loss and endosymbiotic gene transfer (EGT) to the host nucleus, which encodes the majority of the genes for organelle proteomes, as also is the case in primary and secondary plastids. The scope of EGT is not limited to the genes with a function in the plastid, and the nuclear-encoded plastid-targeted genes do not all originate from the endosymbiont (Archibald et al. 2003; Patron et al. 2006; Keeling and Palmer 2008; Reyes-Prieto and Moustafa 2012). The extra layers of endosymbioses, the drastic reduction of endosymbiont, the extra waves of EGTs, and horizontal gene transfers (HGTs) all add to the complexity of these cells, and unraveling their evolutionary histories becomes even more challenging where the symbiotic events are old or at later stages of integration or endosymbiotic reduction. Fortunately, in some instances, like in dinotoms, they are not.

Dinotoms are a small group of dinoflagellates that harbor a tertiary diatom endosymbiont (Horiguchi 2006; Imanian et al. 2010). The endosymbiont is ever-present within the host, and it is transmitted to the daughter cells strictly vertically (Figueroa et al. 2009). Unlike all other secondary and tertiary endosymbionts, however, the dinotom endosymbiont maintains a long list of ancestral characters, including a large nucleus and many mitochondria (Tomas et al. 1973; Jeffrey and Vesk 1976; Horiguchi and Pienaar 1991, 1994; Tamura et al. 2005; Pienaar et al. 2007; Takano et al. 2008). Although the dinoflagellate host seems to have lost its peridinin plastid, or at least photosynthesis, it retains most of its ancestral features, including its large nucleus and mitochondria (Tomas et al. 1973; Jeffrey and Vesk 1976; Horiguchi and Pienaar 1991, 1994; Tamura et al. 2005; Pienaar et al. 2007; Takano et al. 2008). The integration of the well-conserved diatom endosymbiont within a dinoflagellate has generated an exceptional nuclear and cytoplasmic redundancy in dinotoms, which appears to extend to the molecular and genomic levels in the case of mitochondria (Imanian et al. 2012). Also, the plastid genome of dinotoms is more or less unchanged compared with those of free-living diatoms (Imanian et al. 2010). This contrasts starkly with the significant levels of gene loss, gene degradation and/or modifications, major genome rearrangements, and also a gain of transcript editing lacking in haptophyte plastids documented in the tertiary haptophyte-derived plastid genome of *Karlodinium veneficum* (Gabrielsen et al. 2011; Jackson et al. 2013).

**Fig. 1.—:**
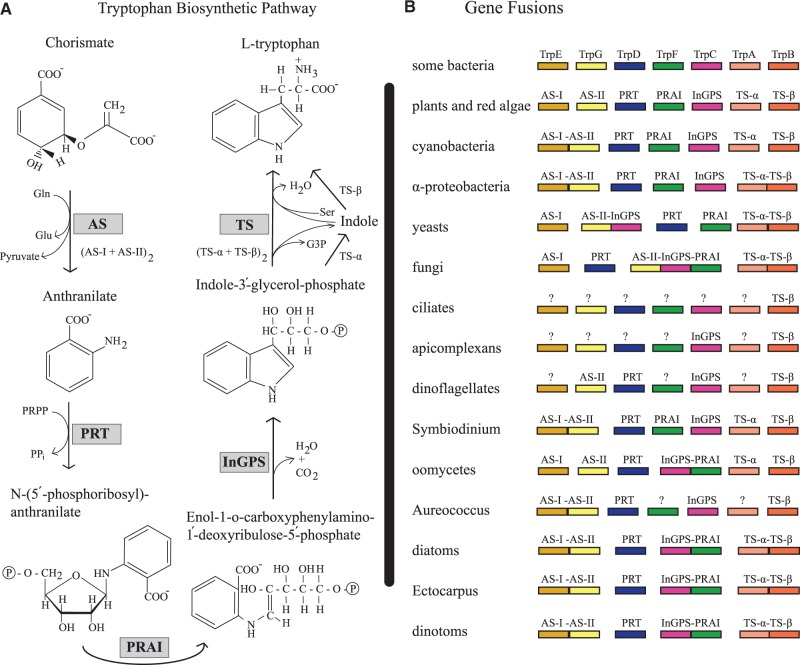
Tryptophan biosynthetic pathway and gene fusions. (*A*) Tryptophan biosynthetic pathway. AS, anthranilate synthase; AS-I and AS-II, components I and II of AS; PRT, anthranilate phosphoribosyltransferase; PRAI, phosphoribosylanthranilate isomerase; InGPS, indoleglycerol phosphate synthase; TS, tryptophan synthase; TS-α and TS-β, α and β subunits of TS; Gln, glutamine; Glu, glutamate; PRPP, 5-phosphoribosyl pyrophosphate; PP_i_, pyrophosphate; G3P, glyceraldehyde-3-phosphate; Ser, serine. (*B*) Gene fusion patterns for the enzymes of tryptophan biosynthesis found in a variety of organisms. The rectangles represent the genes, and connected rectangles represent fused genes. The question mark means that the gene is not found or may not be present in that organism.

These results suggested that genomes of dinotom mitochondria and plastids have evolved under very similar pressures as those in their free-living counterparts, unaffected by the tertiary symbiosis, and, in the case of mitochondria, even allowing for a stable redundancy within the cell. Here, we ask whether an analogous redundancy extends to the nuclear genomes and specifically nucleus-encoded biosynthetic pathways for small molecules. These might be expected to be more easily shared between such intimately associated partners, but currently nothing is known about such pathways. To address this question, we focused on the biosynthesis of tryptophan in three dinotoms, *Durinskia baltica*, *Kryptoperidinium foliaceum*, and *Glenodinium foliaceum*, because tryptophan is an essential amino acid for metazoans and many protists, and its biosynthetic pathway (fig. 1*A*) and regulatory mechanisms are well studied, with all the enzymes been identified in many bacteria, some fungi, plants, and some protists, including two diatoms (Jiroutová et al. 2007). Tryptophan synthesis is a costly affair for the cell, and it is tightly regulated mostly through repression, a feedback inhibition of the first enzyme by tryptophan, and derepression (Crawford 1975; Miozzari et al. 1978). In many bacteria, the genes for these enzymes (TrpA-G) are clustered in varying orders and transcribed together, constituting one or more operons (Crawford 1975). In most eukaryotes, the genes are unlinked (Miozzari et al. 1978). Gene fusions have also played a role in diversifying the genetics behind this pathway, and various gene combinations are reported in both prokaryotes and eukaryotes (Bae and Crawford 1990; Braus 1991; Jiroutová et al. 2007) (see fig. 1*B*).

The first enzyme, anthranilate synthase (AS), is a tetrameric protein composed of two pairs of subunits (components I and II in eukaryotes or TrpG and TrpE in bacteria). The AS synthesizes anthranilate, and the anthranilate phosphoribosyltransferase (PRT or TrpD) transfers a phosphoribosyl group to it. Then, phosphoribosylanthranilate isomerase (PRAI or TrpF) rearranges PRA, and the indoleglycerol phosphate synthase (InGPS or TrpC) closes the ring through a decarboxylation reaction. Finally, tryptophan synthase (TS), also a tetrameric enzyme composed of two pairs of subunits (TS-α or TrpA and TS-β or TrpB), replaces the glycerol phosphate side-chain of indole-3-glycerol-phosphate by the alanyl moiety of an l-serine (fig. 1*A*) (Crawford 1975). Interestingly, two intermediates in this pathway, the anthranilate and indole, permeate freely to most bacterial, fungal, and other cells. This allows certain tryptophan auxotrophs that lack the functional AS or TS-α to grow in the presence of anthranilate or indole, respectively (Crawford 1975).

We searched the databases generated in this study for the transcriptome sequences from three dinotoms, *D. baltica*, *K. foliaceum*, and *G. foliaceum* under two different conditions, light (12:12 light:dark cycle) and dark (after 48 h in the dark), as well as a splice leader (SL) cDNA library for *D. baltica*, and identified all genes related to tryptophan biosynthesis. Phylogenetic analyses show that there are two complete sets of proteins for tryptophan biosynthesis, one set phylogenetically related to diatoms and the other set apparently derived from multiple HGT events to the ancestor of the dinoflagellate host.

## Results and Discussion

We started by searching the available protein and expressed sequence tag (EST) databases (i.e., National Center for Biotechnology Information [NCBI] and the U.S. Department of Energy [DOE] Joint Genome Institute [JGI]) for the enzymes of tryptophan biosynthetic pathway in alveolates, about which little is known. We found the TS-β and InGPS in a few apicomplexans and the TS-β in one ciliate (table 1). TS-β and AS-II, PRT, and InGPS transcripts were also recovered from a few dinoflagellates. We also searched the only available dinoflagellate genome, that of *Symbiodinium minutum* (Shoguchi et al. 2013), and found a few partial copies of AS-I, PRT, PRAI, InGPS, TS-α, TS-β, and, more interestingly, one near complete copy of an AS fusion (components I–II) (table 1). However, phylogenetic analyses revealed that the TS-β and InGPS from the dinoflagellate symbiont of the sea anemone *Anemonia viridis* and all but two of the *S. minutum* sequences likely originated from bacteria (see later and also all the phylogenetic trees are available in newick format in supplementary file S1, Supplementary Material online), implying possible HGTs or contamination. The exceptions in *S. minutum* were AS and TS-β, where it grouped with dinotoms, or other dinoflagellates plus *D. baltica*, respectively (see later and supplementary fig. S1, Supplementary Material online). The presence of the fused AS in *S. minutum* is particularly curious since no other fusion was found in any other alveolate (fig. 1*B*), and the phylogeny suggests these proteins arose relatively early in dinoflagellate diversification. Because apicomplexans and ciliates are among the well-studied eukaryotes with several sequenced genomes, it is unlikely that poor sampling in these taxa could explain the absence of so many genes. It is more likely that they lack all or most of the enzymes for tryptophan synthesis and obtain it, or indole, from their environment (i.e., host or prey). The apparently incomplete and sporadic distribution of these enzymes among the dinoflagellates is more curious, because many are thought to be autotrophic, and suggests that their common ancestor may have also lacked or lost most of the pathway before diversification. On the other hand, the presence of TS-β in many alveolates implies that it is an ancestral trait for this group, and it is possibly selected for as it may allow the heterotrophic members to make tryptophan from indole (fig. 1*A*), which is freely permeable to most cells (Crawford 1975).

**Table 1:** Protein-Coding Genes or Transcripts Involved in Tryptophan Biosynthesis, Found in Alveolates and Stramenopiles

Group	Organism	Protein	Accession
Apicomplexans	*Babesia bovis*	InGPS	154797257
	*B. bovis* T2Bo	InGPS	156085044
	*Cryptosporidium hominis* TU502	TS-B	67583616
	*Theileria annulata*	InGPS	65302611
	*T. annulata* strain Ankara	InGPS	84995456
	*T. orientalis* strain Shintoku	InGPS	403221847
	*T. parva*	InGPS	68351791
	*T. parva* strain Muguga	InGPS	71030386
	*Toxoplasma gondii* ME49	InGPS-domain	211969667
	*To. gondii* ME49	InGPS-domain	237845411
	*To. gondii* VEG	TS-B	221506710
Ciliate	*Paramecium tetraurelia*	TS-B	124430298
Dinoflagellates	*Alexandrium catenella*	TS-B	186958618
	*Al. catenella*	TS-B	186964913
	*Al. minutum*	TS-B	297650291
	*Al. minutum*	TS-B	297651196
	*Al. minutum*	TS-B	297651663
	*Al. ostenfeldii*	TS-B	307932475
	*Al. tamarense*	PRT	40755282
	*Al. tamarense*	PRT	42750557
	*Al. tamarense*	PRT	40759223
	*Al. tamarense*	AS-II	38453270
	*Al. tamarense*	TS-B	42748343
	*Al. tamarense*	TS-B	42750355
	*Karenia brevis*	TS-B	48701842
	*Karlodinium micrum*	AS-II	106843410
	*Oxyrrhis marina*	TS-B	117397467
	*Perkinsus marinus*	AS-II	161749515
	*Symbiodinium minutum*	InGPS	528589502
	*S. minutum*	AS-I	528611714
	*S. minutum*	PRT	528615119
	*S. minutum*	InGPS	528615119
	*S. minutum*	AS-I	528621211
	*S. minutum*	InGPS	528628310
	*S. minutum*	AS-I	528629113
	*S. minutum*	AS-I	528629580
	*S. minutum*	AS-I	528668406
	*S. minutum*	TS-A	528673104
	*S. minutum*	TS-B	528673104
	*S. minutum*	TS-B	528702471
	*S. minutum*	TS-B	528704633
	*S. minutum*	InGPS	528715047
	*S. minutum*	AS-I	528731501
	*S. minutum*	InGPS	528737196
	*S. minutum*	PRAI	524650601
	*S. minutum*	TS-B	524618513
	*S. minutum*	PRT	524609045
	*S. minutum*	AS	524588058
	*S. minutum*	TS-B pseudogene?	524578140
	Symbiont of *Anemonia*	PRT	219235787
	Symbiont of *Anemonia*	PRT	219255915
	Symbiont of *Anemonia*	AS-II	186963243
	Symbiont of *Anemonia*	AS-II	186963244
	Symbiont of *Anemonia*	AS-II	219243193
	Symbiont of *Anemonia*	InGPS	219223170
	Symbiont of *Anemonia*	InGPS	219241272
	Symbiont of *Anemonia*	InGPS	219249362
	Symbiont of *Anemonia*	InGPS	219256877
	Symbiont of *Anemonia*	InGPS	219279257
	Symbiont of *Anemonia*	InGPS	219281076
	Symbiont of *Anemonia*	InGPS	219219692
	Symbiont of *Anemonia*	TS-B	219220957
	Symbiont of *Anemonia*	TS-B	219221386
	Symbiont of *Anemonia*	TS-B	219262239
	Symbiont of *Anemonia*	TS-B	219275118
	Symbiont of *Anemonia*	TS-B	219279244
Pelagophyte	*Aureococcus anophagefferens*	AS	323450740
		AS	323451037
		PRT	323448946
		InGPS	323451133
		TS-B	323453341
		PRAI-UPRT-GTPCH	323450452
Phaeophyte	*Ectocarpus siliculosus*	AS	298711406
		InGPS	298712952
		InGPS-PRAI	299116131
		PRT	298710515
		TS	299472124

Note.—Protein abbreviations: AS, anthranilate synthase components I and II; AS-II, anthranilate synthase component II; InGPS-PRAI, Indoleglycerolphosphate synthase (InGPS) and phosphoribosylanthranilate isomerase (PRAI) fusion; PRAI-UPRT-GTPCH, PRAI and urasilphosphoribosyl transferase and GTP cyclohydrolase N terminal fusion; TS, tryptophan synthase; TS A and B or α and β subunits; TS-B, TS-B or β subunit.

**Fig. 2.—:**
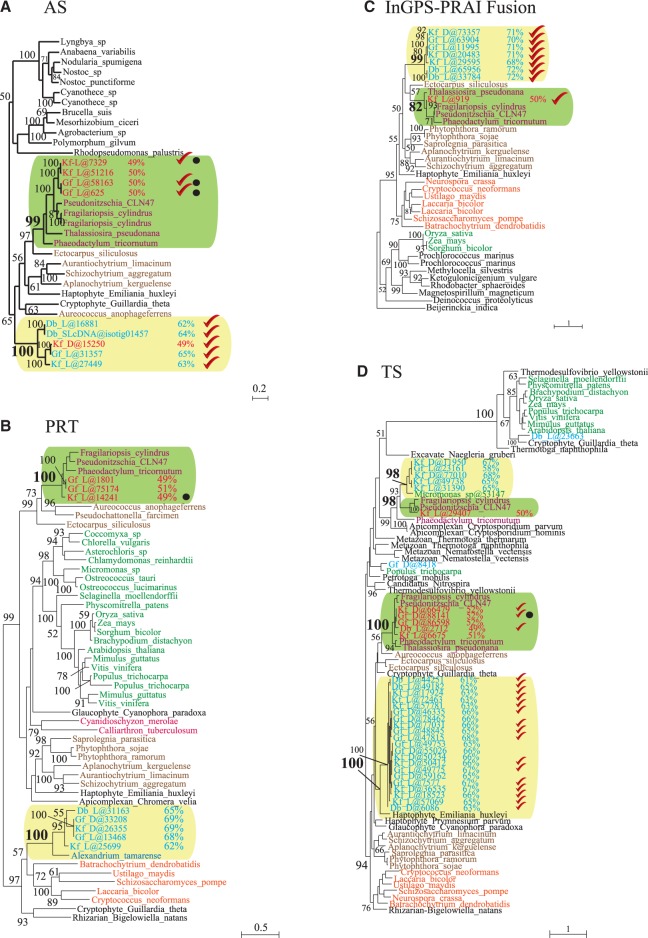
The maximum likelihood trees for the enzymes of the tryptophan biosynthetic pathway in dinotoms. (*A*) Anthranilate synthase (AS) phylogeny, partial tree. (*B*) Anthranilate phosphoribosyltransferase (PRT), partial tree. (*C*) Indole-3-glycerol-phosphate synthase and phosphoribosylanthranilate isomerase fusion (InGPS-PRAI) phylogeny. (*D*) Tryptophan synthase (TS) phylogeny, partial tree. Numbers at the nodes indicate the bootstrap support ≥ 50 for the majority of the nodes. The dinotom clades are highlighted with boxes in green (with diatoms) and cream. The numbers next to dinotom taxa indicate the GC content of their protein-coding transcripts. The checkmarks indicate the fusion proteins in dinotoms. The black dots denote the presence of an SP as predicted by SignalP 3.0 (Bendtsen et al. 2004). The dinotom sequences with a low or high GC content are shown in red or turquoise fonts, respectively. Some major groups are also color coded: diatoms in purple font; other stramenopiles in brown; streptophytes and green algae in green; red algae in scarlet; dinoflagellates in blue; and fungi in orange. All other groups are in black font, and with the exception of prokaryotes, the name of the group appears before the species name. The accession numbers are given in the supplementary file S1, Supplementary Material online. Db-D/L, *Durinskia baltica* dark/light; Kf-D/L, *Kryptoperidinium foliaceum* dark/light; Gf-D/L, *Glenodinium foliaceum* dark/light.

To see how the dinotoms fit into this picture, total mRNA sequence data for each dinotom was searched for enzymes involved in tryptophan biosynthesis. Unlike other alveolates, we recovered transcripts corresponding to the complete pathway: the AS (components I and II), PRT, InGPS-PRAI fusion, and TS (α and β subunits) (supplementary file S1, Supplementary Material online). With the exceptions of the AS and PRT in *D. baltica*, we found multiple copies of all the transcripts in the dinotoms (for a total of 73 distinct cDNAs). From the *D. baltica* SL cDNA sequence data, only one copy of the AS (components I and II) was recovered. The protein alignments of these sequences with their respective homologs in other eukaryotes and/or prokaryotes showed that most encoded the entire mature protein sequence, but it is not clear if they represent full-length transcripts because few dinoflagellate SL was found at the 5′-end of their cDNAs (like most transcripts from these libraries), which suggests the possibility that they are missing at least part of the 5′-untranslated regions. Because tryptophan biosynthesis has been reported to take place in the diatom plastid (Jiroutová et al. 2007), we sought evidence for signal and transit peptides in the dinotom sequences. Only the *G. foliaceum* AS and TS and the *K. foliaceum* AS and PRT were predicted to have a signal peptides (SPs, all within diatom clades, marked by black dots in fig. 2), and none were predicted to encode transit peptides. These predictions are not always accurate, and many transcripts are truncated, so we only conclude that it is possible that some or all the diatom-derived proteins are targeted to the diatom plastid.

We reconstructed phylogenetic trees for the identified proteins involved in tryptophan biosynthesis in dinotoms. The dinotom sequences fall into two distinct clades in all four trees (fig. 2). In all phylogenies, at least one dinotom is represented in a strongly supported diatom clade (fig. 2). In the PRT phylogeny, the second dinotom clade includes the dinoflagellate *Alexandrium tamarense* at its base (100% support: fig 2*B*), whereas in the InGPS-PRAI tree, the second group of dinotom sequences falls within a larger stramenopile clade, sister to (no support) but distinct from the strongly supported diatom subclade (fig. 2*C*). The TS phylogeny is more complex as it includes both the TS (α-β-fusion, checkmarked in fig. 2*D*) and TS-β sequences, both of which are present in both dinotoms and diatoms. Dinotom sequences therefore branch in four distinct clades: two consisting of TS fusion proteins and two consisting of TS-β proteins. One dinotom TS fusion clade is nested within diatoms, and the other branches with the haptophyte *Emiliania huxleyi* at its base, both with strong support (fig. 2*D*). One *K. foliaceum* TS-β also branches with the diatoms (100% support) and the other forms a sister group to this clade, with the green alga *Micromonas* (fig. 2*D*).

**Fig. 3.—:**
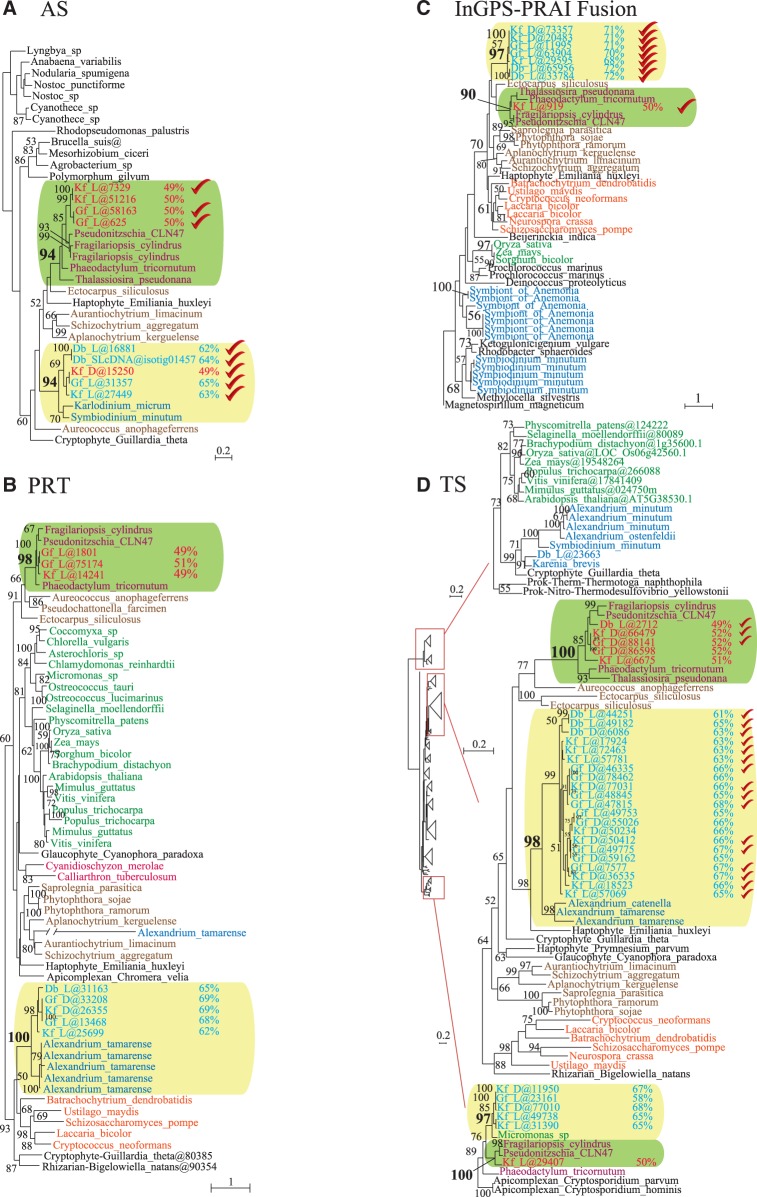
The maximum likelihood trees for the enzymes of the tryptophan biosynthetic pathway in dinoflagellates. (*A*) Anthranilate synthase (AS) phylogeny, partial tree. (*B*) Anthranilate phosphoribosyltransferase (PRT). (*C*) Indole-3-glycerol-phosphate synthase and phosphoribosylanthranilate isomerase fusion (InGPS-PRAI) phylogeny. (*D*) Tryptophan synthase (TS) phylogeny, partial tree. Numbers at the nodes indicate the bootstrap support ≥ 50 for the majority of the nodes. The dinotom clades are highlighted with boxes in green (with diatoms) and cream (with dinoflagellates). The dinotom sequences with a low or high GC content are shown in red or turquoise fonts, respectively. Some major groups are also color coded: diatoms in purple font; other stramenopiles in brown; streptophytes and green algae in green; red algae in scarlet; dinoflagellates in blue; and fungi in orange. All other groups are in black font, and with the exception of prokaryotes, the name of the group appears before the species name. The accession numbers are given in the supplementary file S1, Supplementary Material online. Db-D/L, *Durinskia baltica* dark/light; Kf-D/L, *Kryptoperidinium foliaceum* dark/light; Gf-D/L, *Glenodinium foliaceum* dark/light.

The additional phylogenetic analyses of the identified dinoflagellate proteins for tryptophan synthesis (supplementary file S1, Supplementary Material online) did not change the overall topology or support for the trees significantly especially for dinotom clades (compare figs. 2 and 3). As noted earlier, TS-β and InGPS trees for the symbiont of *A. viridis* and most of the *S. minutum* sequences grouped with bacteria and are most likely contaminants or recent HGTs (fig. 3 and supplementary fig. S1, Supplementary Material online). In contrast, the remaining dinoflagellate sequences for AS-II, PRT, and TS-β all branched with the nondiatom dinotom sequences (fig. 3 and supplementary fig. S1, Supplementary Material online). This included AS sequences from *Karlodinium micrum*, PRT sequences from *Al. tamarense*, and TS sequences from *Al. tamarense* and *A. catenella*, which branched specifically with the TS fusion clade (fig. 3).

**Fig. 4.—:**
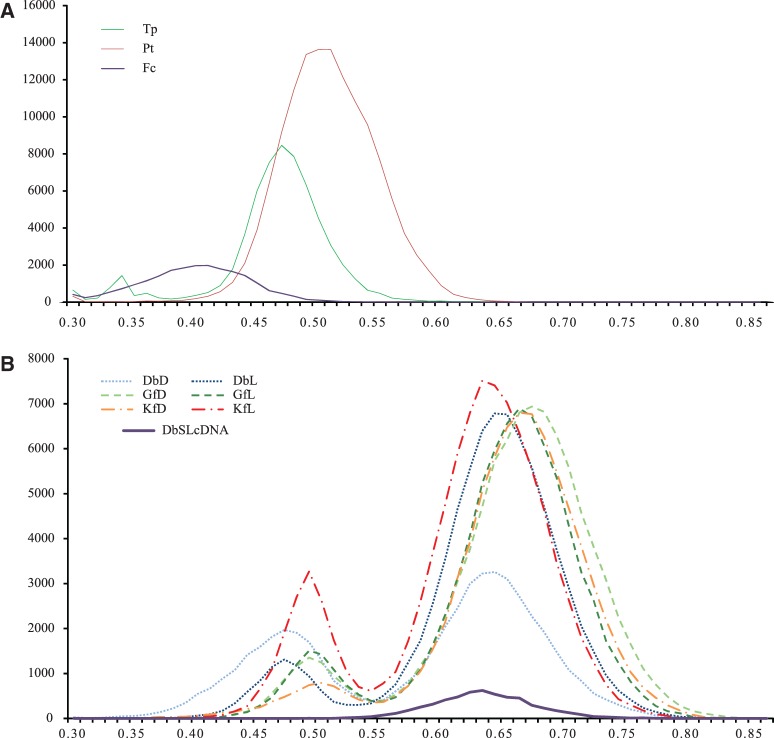
The distribution of the GC content of the diatom and dinotom sequences. (*A*) The distribution of the GC content of all the EST sequences > 150 bp available for three diatoms, downloaded from the National Center for Biotechnology Information EST database on December 4, 2013. Fc, *Fragilariopsis cylindrus*; Pt, *Phaeodactylum tricornutum*; Tp, *Thalassiosira pseudonana*. (*B*) The distribution of the GC content of the dinotom total mRNA and SL cDNA sequences. The *x* axis shows the GC content, and the *y* axis the number of sequences. DbD, *Durinskia baltica* dark sample; DbL, *D. baltica* light sample; GfD, *Glenodinium foliaceum* dark sample; GfL, *G. foliaceum* light sample; KfD, *Kryptoperidinium foliaceum* dark sample; KfL, *K. foliaceum* light sample; DbSLcDNA, *D. baltica* SL cDNA library.

Interestingly, all the dinotom proteins that clustered with those of diatoms in the phylogenetic trees (fig. 2) had cDNAs with low GC content (∼50%), similar to that of the nuclear genome of two diatoms, *Phaeodactylum tricornutum* (48.9%) and *Thalassiosira pseudonana* (46.9%), or their coding sequences (50.0% and 48.0%, respectively) (Armbrust et al. 2004; Bowler et al. 2008) (see also fig. 4*A*). In contrast, almost all the dinotom cDNAs for the proteins in the nondiatom clade had significantly higher GC content. The distribution of the GC content of the dinotom mRNA sequences also shows two distinct peaks, one low (∼50%) and the other high (∼65%), whereas there is only one peak (∼65%) for that of the sequences in *D. baltica* SL cDNA library, which is enriched in dinoflagellate sequences (fig. 4*B*). This is also consistent with early observations based on cloned genes (McEwan and Keeling 2004). Taking this together with the phylogenies all suggest that the dinotom proteins with low-GC-content cDNA that branched strongly with the diatoms are encoded in the nucleus of the diatom endosymbiont, whereas the proteins with high-GC-content cDNA that branched with other dinoflagellates are encoded in the nucleus of the dinoflagellate host.

It is noteworthy that none of these dinoflagellate host clades was demonstrably related to other alveolates, even when other alveolates do possess the genes (most are absent from apicomplexans and ciliates). This suggests that the dinoflagellate host genome acquired their genes independently. The position of this clade is not well supported in AS and InGPS trees, and branches with a clade composed of various distantly related eukaryotes in PRT phylogeny, so there is no obviously single “source” for these genes, but there is a weak association with stramenopiles in AS, InGPS, and TS-β.

As to why such redundancy persists in dinotoms, we have suggested earlier (Imanian and Keeling 2007) that the membrane separating the diatom endosymbiont from the dinoflagellate host, derived perhaps from the cell membrane of the diatom (Eschbach et al. 1990), may act as a barrier to integration, due to lack of transporters, for example. In this case, we do not know how readily diffusible amino acids are between the two partners, but the apparent redundancy in the expression of all genes needed to synthesize tryptophan suggests perhaps that neither the amino acid nor its intermediates are easily exchanged. Complete genomes from both partners and direct biochemical assays of how nutrients are or are not exchanged will certainly clarify this in the future.

Overall, we found two complete and distinct sets of transcripts for the enzymes of tryptophan biosynthetic pathway in dinotoms, one originating from the diatom endosymbiont and the other from the dinoflagellate ancestor of the dinotom host, which acquired them through HGT (figs. 2, 3, and supplementary fig. S1, Supplementary Material online). Although additional investigation at the protein level is needed to further characterize tryptophan metabolism and its likely redundancy in dinotoms, our results indicate, for the first time, that the unique redundancy observed in dinotom mitochondrial genomes and transcriptomes (Imanian et al. 2012) extends also to their nuclear genomes and gene products expressed in the cytosol.

## Materials and Methods

### Cultures, Media, Growth, and Harvest Conditions

Cultures of *D. baltica* (*Peridinium balticum*) CSIRO CS-38, *K. foliaceum* CCMP 1326, and *G. foliaceum* CCAP 1116/3 were obtained from the CSIRO Microalgae Supply Service (CSIRO Marine and Atmospheric Research Laboratories, Tasmania, Australia), the Provasoli-Guillard National Center for Culture of Marine Phytoplankton (West Boothbay Harbor, ME), and Culture Collection of Algae and Protozoa (CCAP SAMS Research Services Ltd. Scottish Marine Institute, OBAN, Scotland, UK), respectively. *Durinskia baltica* culture was maintained in GSe medium at 22 °C in 12:12 light:dark cycle (light samples) and after 48 h in the dark (Dark samples), whereas *K. foliaceum* and *G. foliaceum* cultures were maintained in F/2-Si medium under the same conditions.

### Nucleic Acid Extractions, Purification, and the SL cDNA and Poly-A Library Construction, Sequencing, and Assembly

Exponentially growing cells were collected and ground as described elsewhere (Imanian et al. 2007). Cell lysis, nucleic acid extractions, precipitations, and purifications were performed as described earlier (Imanian et al. 2010). The total RNA was cleaned up after DNase treatment (RNeasy MinElute Cleanup kit; Qiagen, Mississauga, ON), and poly-A RNA was purified from 25 µg of cleaned-up total RNA (Oligotex mRNA Mini Kit; Qiagen, Mississauga, ON). Approximately 500 ng of poly-A RNA from *D. baltica* were used as template for constructing first and second strand cDNA (Just cDNA Double Stranded cDNA Synthesis kit; Agilant Technologies Canada, Mississauga, ON) with a dinoflagellate-specific SL primer (5′-CCGTAGCCATTTTGGCTCAAG-3′). The resulting double-stranded cDNA sample was amplified through polymerase chain reaction (PCR) and/or long-range PCR with the SL primer in conjunction with the random 9mer primers. The amplified cDNA sample was purified (QIAquick PCR Purification kit; Qiagen, Mississauga, ON) and reamplified once more through PCR and/or long-range PCR.

The amplified SL cDNA of *D. baltica* was sequenced using massively parallel GS-FLX DNA pyrosequencing (Roche 454 Life Sciences, Branford, CT), which was carried out at the Génome Québec Innovation Centre. This pyrosequencing produced a total of 553,695 reads with an average length of 351 bp. The reads were assembled de novo using gsAssembler 2.5p1 (formerly known as Newbler), edited, and reassembled with CONSED 23 (Gordon et al. 1998; Gordon 2004) to remove the misaligned reads. The final assembly contained 65% of all the reads that were assembled into 5,625 large contigs. This Transcriptome Shotgun Assembly project has been deposited at DDBJ/EMBL/GenBank under the accession GAAT00000000. The version described in this paper is the first version, GAAT01000000.

The library preparation, sequencing, assembling, and annotating the poly-A transcriptome of the three dinotoms were performed by and at the National Centre for Genome Resources (see supplementary file S2, Supplementary Material online).

### Phylogenetic Analyses of the Enzymes of Tryptophan Biosynthesis Pathway in Dinotoms

The protein sequences for the tryptophan biosynthetic pathway in dinotoms and dinoflagellates were identified (see supplementary file S2, Supplementary Material online) and used as queries in a BlastP (Altschul et al. 1990) homology search with an *e* value < 1e − 5 against the protein collections from complete genomes and EST databases (see supplementary file S1, Supplementary Material online). The sequence retrieval, alignment, and tree reconstruction were conducted as described elsewhere (Burki et al. 2012) with a few modifications (see supplementary file S2, Supplementary Material online). RAxML 7.2.8 (Stamatakis 2006) was run to reconstruct the phylogenetic trees, with LG substitution matrix + Γ4 + F evolutionary model with 100 bootstrap replicates. PhyloSort (Moustafa and Bhattacharya 2008) was used to cluster the repetitive phylogenetic trees for the queries with multiple paralogs. The 49 reconstructed phylogenies divided into four clusters corresponding to the four proteins (AS, PRT, InGPS-PRAI, and TS). Then, all the clustered trees were individually and manually examined. A representative phylogeny for each cluster is shown in figure 2. Because very few of the identified dinoflagellate proteins for tryptophan synthesis met the length criterion in our analyses (50% of the total length of the alignments), they were analyzed separately once as new queries and once they were added to their dinotom homologs and their corresponding hits (fig. 3), following the same procedure described earlier. In some cases, we noted that *K. foliaceum* and *G. foliaceum* data sets included pairs of highly similar paralogs. These two organisms are extremely closely related (perhaps strains of a single species), so we cannot distinguish between cross contamination between two samples at the sequencing stage (which is common with Illumina sequencing) and closely related copies of the gene. In all such cases, however, other distinct copies of the gene also existed in both data sets, so neither possibility affects the conclusions.

### GC Content Calculations and Targeting Signal Predictions

The GC content of all the sequences were calculated using GEECEE from the EMBOSS package (Rice et al. 2000). The presence/absence of the 5′-end of transcripts was determined after aligning them with their best eukaryotic and/or prokaryotic homologs. SignalP 3.0 (Bendtsen et al. 2004) with NN option and ChloroP (Emanuelsson et al. 1999) were used to search for an SP and plastid transit peptide, respectively.

## Supplementary Material

Supplementary files S1 and S2 and figure S1 are available at Genome Biology and Evolution online (http://www.gbe.oxfordjournals.org/).

Supplementary Data
