# Supplementary material for: Horizontal Gene Transfer and Redundancy of Tryptophan Biosynthetic Enzymes in Dinotoms
Source: Genome Biol Evol. 2014 Jan 21;6(2):333–43. doi: 10.1093/gbe/evu014 (PMC3942023; doi:10.1093/gbe/evu014)
Supplement: Supplementary Data [file supp_6_2_333__index.html]

Horizontal gene transfer and redundancy of tryptophan biosynthetic enzymes in dinotoms — Horizontal Gene Transfer and Redundancy of Tryptophan Biosynthetic Enzymes in Dinotoms — Supplementary Data 

# Horizontal Gene Transfer and Redundancy of Tryptophan Biosynthetic Enzymes in Dinotoms

## Supplementary Data

files

**Files in this Data Supplement:**

- Supplementary Data - zip file
