# Supplementary material for: Horizontal Gene Transfer and Redundancy of Tryptophan Biosynthetic Enzymes in Dinotoms
Source: Genome Biol Evol. 2014 Jan 21;6(2):333–43. doi: 10.1093/gbe/evu014 (PMC3942023; doi:10.1093/gbe/evu014)
Supplement: Supplementary Data [file supp_evu014_suppl_data.zip › Supplementary_File_S2.docx]

**Library preparation, sequencing, assembling and annotating the transcriptome of the three dinotoms performed by and at the National Centre for Genome Resources (NCGR)**

Invitrogen Qubit Q32855 and the Agilent 2100 Bioanalyzer were used to quantify and assess the RNA quality, respectively. The Illumina’s TruSeq RNA Sample Preparation Kit was used to make the libraries from the 2 µg of cleaned RNA. The average insert size of each library ranged from 250 to 350 bp. Libraries were sequenced on the Illumina HiSeq 2000 to obtain 2 x 50 bp (paired-end) reads. Over 2 Gbp of sequence was generated per library.

Transcriptome assembly was carried out using NCGR’s internal pipeline called BPA1.0 (Batch Parallel Assembly version 1.0). Sequence reads were preprocessed using SGA preprocess (Simpson and Durbin 2012) for quality trimming (swinging average) at Q15. Reads less than 25 nt after trimming were discarded. Preprocessed sequence reads were assembled into contigs with ABySS (Simpson et al. 2009) v. 1.3.0, using 20 unique kmers between k=26 and k=50. ABySS was run requiring a minimum kmer coverage of 5, bubble popping at > 0.9 branch identity with the scaffolding flag enabled to maintain contiguity for divergent branching. Paired-end scaffolding was performed on each kmer. Sequence read pairing information was used in GapCloser (Li et al. 2008) v. 1.10 to walk in on gaps created during scaffolding in each individual kmer assembly. Contigs from all gap-closed kmer assemblies were combined. The OLC (overlap layout consensus) assembler miraEST (Chevreux et al. 2004) was used to identify minimum 100 bp overlaps between the contigs and assemble larger contigs, while collapsing redundancies. BWA (Li and Durbin 2009) was used to align sequence reads back to the contigs. Alignments were processed by SAMtools mpileup (http://samtools.sourceforge.net) to generate consensus nucleotide calls at positions where IUPAC bases were introduced by miraEST (Chevreux et al. 2004), and read composition showed a predominance of a single base. In an attempt to remove incomplete sequences, the consensus contigs were filtered at a minimum length of 150 nt to produce the final set of contigs.

Coding sequences were predicted using ESTScan (Iseli et al. 1999; Lottaz et al. 2003) with a Bacillariophyta scoring matrix. Sequence reads were aligned back to the nucleotide motifs of the predicted coding sequences using BWA (Li and Durbin 2009). Peptide predictions over 30 amino acids in length were annotated. BLASTp (Altschul et al. 1990) was used to generate hits against the UniProtKB/Swiss-Prot database. Protein sequences were also functionally characterized using HMMER3 (Zhang and Wood 2003) against Pfam-A (Finn et al. 2008), TIGRFAM (Haft et al. 2001), and SUPERFAMILY (Gough et al. 2001) databases.

**Identification of the enzymes of tryptophan biosynthesis pathway in dinotom and dinoflagellate transcriptome datasets**

ORFPredictor (Min et al. 2005) was used to translate the *D. baltica* SL cDNA sequences. These peptide sequences were added to the peptide sequences of the three dinotoms under both conditions (Light and Dark) (see above). A subset of the NCBI non-redundant (nr) database (http://www.ncbi.nlm.nih/BLAST) that included all the sequences for the proteins in the tryptophan biosynthetic pathway downloaded on 2013/03/31. Also all the dinoflagellate EST sequences were downloaded on the same date from the NCBI EST database. The dinotom proteins and the dinoflagellate EST sequences were initially used as a query in a BLASTP and a BLASTX homology search (Altschul et al. 1990), respectively, with a cut-off e-value of 1e-05 against the tryptophan biosynthetic protein database mentioned above. The dinoflagellate hit sequences were translated to peptides using ORFpredictor (Min et al. 2005). The dinotom and dinoflagellate hit sequences then were used as queries in a new BLASTP homology searches against the entire NCBI nr database and the NCBI’s Conserved Domain Database (Marchler-Bauer et al. 2010) with the same cut-off e-value. The sequences whose first 5-10 hits were to an enzyme in the tryptophan biosynthetic pathway and/or had their respective conserved domain(s) were identified as the enzymes of this pathway in dinotoms and dinoflagellates.

**Phylogenetic analyses of the dinotom proteins for tryptophan biosynthetic pathway**

The following modifications were made in sequence retrieval, alignment and tree reconstruction methods described in (Burki et al. 2012). CDHIT (Li and Godzik 2006) was utilized to remove redundant sequences and close paralogues from each protein database to simplify interpretations of the resulting phylogenetic trees (with 85% identity threshold for clustering). The blast output file was parsed with a strict e-value threshold of 1e-25 to reduce the number of distantly related paralogues and to generate multiple fasta files including each protein query and its corresponding hits. The sequences in each file were aligned using MAFFT (Katoh and Toh 2008) with the fftnsi option, and alignment positions were selected and sites containing more than 10% of gaps were removed using TRIMAL (Capella-Gutiérrez et al. 2009). The alignment files with fewer than 5 species or when the query sequences were shorter than 50% of the total length of the alignments were discarded at this stage.

**References**

Altschul SF, Gish W, Miller W, Myers EW, Lipman DJ. 1990. Basic local alignment search tool. J. Mol. Biol. 215:403–410.

Burki F et al. 2012. Re-evaluating the Green versus Red Signal in Eukaryotes with Secondary Plastid of Red Algal Origin. Genome biol. evol. 4:evs049.

Capella-Gutiérrez S, Silla-Martínez JM, Gabaldón T. 2009. trimAl: a tool for automated alignment trimming in large-scale phylogenetic analyses. Bioinformatics (Oxford, England). 25:1972–3.

Chevreux B et al. 2004. Using the miraEST assembler for reliable and automated mRNA transcript assembly and SNP detection in sequenced ESTs. Genome Res. 14:1147–59.

Finn RD et al. 2008. The Pfam protein families database. Nucleic Acids Res. 36:D281–8.

Gough J, Karplus K, Hughey R, Chothia C. 2001. Assignment of homology to genome sequences using a library of hidden Markov models that represent all proteins of known structure. J. Mol. Biol. 313:903–19.

Haft DH et al. 2001. TIGRFAMs: a protein family resource for the functional identification of proteins. Nucleic Acids Res. 29:41–3.

Iseli C, Jongeneel C V, Bucher P. 1999. ESTScan: a program for detecting, evaluating, and reconstructing potential coding regions in EST sequences. In Proc. Int. Conf. Intell. Syst Mol. Biol. 138–48.

Katoh K, Toh H. 2008. Recent developments in the MAFFT multiple sequence alignment program. Brief. bioinform. 9:286–98.

Li H, Durbin R. 2009. Fast and accurate short read alignment with Burrows-Wheeler transform. Bioinformatics (Oxford, England). 25:1754–60.

Li R, Li Y, Kristiansen K, Wang J. 2008. SOAP: short oligonucleotide alignment program. Bioinformatics (Oxford, England). 24:713–4.

Li W, Godzik A. 2006. Cd-hit: a fast program for clustering and comparing large sets of protein or nucleotide sequences. Bioinformatics (Oxford, England). 22:1658–9.

Lottaz C, Iseli C, Jongeneel C V., Bucher P. 2003. Modeling sequencing errors by combining Hidden Markov models. Bioinformatics. 19:ii103–ii112.

Marchler-Bauer A et al. 2010. CDD: a Conserved Domain Database for the functional annotation of proteins. Nucleic Acids Res. 39:D225–9.

Min XJ, Butler G, Storms R, Tsang A. 2005. OrfPredictor: predicting protein-coding regions in EST-derived sequences. Nucleic Acids Res. 33:W677–80.

Simpson JT et al. 2009. ABySS: a parallel assembler for short read sequence data. Genome Res. 19:1117–23.

Simpson JT, Durbin R. 2012. Efficient de novo assembly of large genomes using compressed data structures. Genome Res. 22:549–56.

Zhang Z, Wood WI. 2003. A profile hidden Markov model for signal peptides generated by HMMER. Bioinformatics (Oxford, England). 19:307–8.
